# Multi-Walled Carbon-Nanotube-Reinforced PMMA Nanocomposites: An Experimental Study of Their Friction and Wear Properties

**DOI:** 10.3390/polym15132785

**Published:** 2023-06-22

**Authors:** Vijay Patel, Unnati Joshi, Anand Joshi, Blessing Kudzai Matanda, Kamlesh Chauhan, Ankit D. Oza, Diana-Petronela Burduhos-Nergis, Dumitru-Doru Burduhos-Nergis

**Affiliations:** 1 Department of Mechanical Engineering, Parul University, Vadodara 391760, Gujarat, India; vijaypatel.2612@gmail.com (V.P.); unnatiajoshi@gmail.com (U.J.); 210305208014@paruluniversity.ac.in (B.K.M.); 2 Department of Mechatronics Engineering, Parul University, Vadodara 391760, Gujarat, India; 3 Department of Mechanical Engineering, Charusat University, Anand 388421, Gujarat, India; kamleshchauhan.me@charusat.ac.in; 4 Department of Computer Sciences and Engineering, Institute of Advanced Research, Gandhinagar 382426, Gujarat, India; ankit.oza@iar.ac.in; 5 Faculty of Materials Science and Engineering, Gheorghe Asachi Technical University of Iasi, 700050 Iasi, Romania; diana.burduhos@tuiasi.ro

**Keywords:** nanocomposite, tribology, scanning electron microscopy, carbon nanotubes

## Abstract

This manuscript presents an experimental investigation of the friction and wear properties of poly (methyl methacrylate) (PMMA) nanocomposites reinforced with functionalized multi-walled carbon nanotubes (MWCNTs). The aim of this study is to evaluate the potential of MWCNTs as a reinforcement material for enhancing the tribological performance of PMMA. Three types of multi-walled carbon nanotubes, i.e., pristine, hydroxyl functionalized, and carboxyl functionalized, were utilized in this study. The nanocomposite samples were prepared by dispersing varying concentrations of MWCNTs (0.1 wt.%, 0.5 wt.%, and 1 wt.%) within the PMMA matrix via a 3D mixing approach, followed by injection molding/compression molding. The resulting nanocomposite films were characterized using scanning electron microscopy (SEM) to observe the dispersion of MWCNTs within the PMMA matrix. The friction and wear tests were conducted using a pin-on-disk tribometer under dry sliding conditions. The effects of functionalization and MWCNT content on the tribological behaviors of the nanocomposites were analyzed. The nanocomposites exhibited lower friction coefficients and reduced wear rates compared to pure PMMA. The lowest friction coefficient and wear rate were achieved at an optimum MWCNT loading of 0.5 wt.%. It was further revealed that the amount of MWCNT reinforcement, average load, and track diameter significantly affect the coefficient of friction (COF) and rate of wear. The COF and wear rate are best at a filler loading of 0.5 wt.%, a 20 Kg load, and 90 mm. The improved tribological performance of the MWCNT-reinforced PMMA nanocomposites can be attributed to the effective transfer of load between the MWCNTs and the PMMA matrix, as well as the reinforcement effect of the MWCNTs. The MWCNTs acted as reinforcing agents, enhancing the mechanical properties and wear resistance of the nanocomposites.

## 1. Introduction

In response to global concerns, researchers, academicians, and companies have recently turned their attention to polymer nanocomposite materials and their potential uses in the aerospace, automotive, electronics, military, and infrastructure sectors. A polymer nanocomposite material’s tribological properties are crucial to its reliable operation as an effective alternative to metallic materials due to its improved optical, mechanical, and electrical properties [1]. Artificial biological materials, medical equipment, and industrial elements like gears and bearings are all regularly subject to wear and friction effects. As matrices, polymers offer numerous advantages over other materials, including lower friction coefficients, superior chemical resistance, high mechanical strength, lighter weight, and improved wear resistance [2]. As a result, researchers are focusing on finding new polymer–filler combinations that can be used to make composite materials with improved functional properties [3]. Whether combining nanoparticles, carbon nanotubes (CNTs), graphene oxide (G.O.), graphene (Gr), nanoclays, etc., with various matrix materials can provide innovative materials with improved mechanical performances has been investigated [4,5,6,7]. Polyetheretheretherketone (PEEK), as reported by Baligidad et al., has found extensive use as a metal substitute in the medical field [8]. Graphene oxide (G.O.)/carbon nanotube (CNT)/polypropylene (P.P.) nanocomposite materials were created using an epoxy resin (E.P.) via a hand lay-up approach, which improved their mechanical characteristics and microstructures. All the advanced composite materials showed the expected tensile strength, flexural modulus, and flexural strength values. Significant quantities of functionalized G.O., P.P., and CNTs reduced friction and wear in the nanocomposites [9].

Sudeepan et al. examined the mechanical and tribological characteristics of acrylonitrile–butadiene–styrene (ABS), utilizing a range of clay filler mixing ratios. Up to a certain point, the mechanical properties improved with the addition of more filler, but after that point, they worsened. The results showed that filler content, normal load, and sliding speed significantly impact the friction coefficient and specific wear rate when the friction coefficients and wear rates of polymer composites were investigated [10]. Campos-Sanbria et al. created poly (methyl methacrylate)/hydroxyapatite (PMMA/HAp) nanocomposites. Three different benzoyl peroxide (PBO) mixing ratios were used to develop these nanocomposites. The results showed that the PMMA, Hap, and PBO nanocompositse with better mixing ratios had the most desirable properties. The friction coefficients reduced as the proportion of PBO increased, enabling the fastest rate of wear conceivable [11]. Anandarao et al. investigated the wear and friction properties of polytetrafluoroethylene (PTFE), graphite, and molybdenum composite materials.

Compression molding and the powder metallurgy concept were used to prepare the specimen. Six different mixing ratios of molybdenum in PTFE and graphite were used. The influences of filler, load, and sliding distance were evaluated as a part of the trial results, and the specimen was found to have a lower wear rate than pristine PTFE. Also, it was concluded that the wear rate is directly proportional to the load and the sliding velocity [12]. Daniel and Liu presented a comprehensive investigation of graphene and carbon fibers reinforcing polyimide and the remarkable features of both materials. The results of a study that compared the tribological properties of graphene/polyimide and carbon fibers/polyimide were analyzed. The findings showed a considerable improvement in the friction and wear properties of the material made with graphene and nanofiller [13]. In their article, Chan et al. discussed the latest developments in evaluating the tribological properties of polymer nanocomposites. The paper also discussed the effect of the mixing ratio and the type of nanofiller and its functionalization on the friction and wear of prepared nanocomposites [14]. Jesthi et al. carried out a design experiment using the response surface methodology (RSM) and investigated the mechanical and wear properties of a polymer composite reinforced with glass and carbon fiber [15].

Petre et al. [16] conducted an experimental investigation on the tribological properties of the polymeric material known as Relamid, which is utilized in the production of functional components for machines. According to the findings, the rate at which the material wears relies on the working parameters of the device, such as the speed, sliding distance, and load. It has also been discovered that the sliding rate affects the coefficient of friction, causing it to decrease as the sliding speed increases. Nanocomposites were created by Ferreira et al. using high-molecular-weight polyethylene (HMWPE) and a trace amount of multilayer graphene oxide (mGO). In light of their findings, they concluded that the nanocomposites’ mechanical and tribological properties were significantly improved compared to those of ultra-high-molecular-weight polyethylene (UHMWPE). Both differential scanning calorimetry (DSC) and transmission electron microscopy (TEM) were used in the morphological investigation that was conducted [17]. PPS-based composites loaded with PTFE and nanoparticles (SiO_2_, Al_2_O_3_, and CuFe_2_O_4_) have had some of their mechanical and tribological properties examined. It has been established that adding nanoparticles to a combination of “PPS + 10 wt.% PTFE” is a successful method that decreases the coefficient of friction by a factor of two and improves the overall performance of the combination. The ability of PPS-based ternary composites to adhere to the transfer layers on metal and ceramic counterpart surfaces was utilized in calculating the wear rate of nanoparticles of different natures (SiO_2_, Al_2_O_3_, and CuFe_2_O_4_) [18]. A brief summarization of the previous literature is presented in Table 1.

Tribology is the study of the interactions that occur between surfaces that are moving in relative motion. This research branch addresses some of today’s most pressing concerns, including friction, wear, and lubrication. The dispersion states of carbon nanotubes (CNTs) can indeed significantly affect their tribological properties. Achieving a good dispersion of CNTs within a matrix material is important for ensuring an efficient load transfer, enhancing mechanical properties, and optimizing tribological performance. Surface modification techniques can be employed to enhance the dispersion of CNTs. The functionalization of CNTs with chemical groups can improve their compatibility with the matrix material, reducing agglomeration tendencies and promoting better dispersion [28,29]. Over the past few decades, polymeric materials have substantially usurped the function of conventional engineering materials in tribological applications. This paper investigates in depth several crucial factors that significantly impact tribal performances. These factors include test settings, the ratio of matrix to nanofiller, and the operating environment. In addition, it elucidates the numerous methods through which fillers can be incorporated into fundamental matrices to increase their properties.

This manuscript presents a novel investigation into the friction and wear properties of PMMA (polymethyl methacrylate) nanocomposites reinforced with functionalized multi-walled carbon nanotubes (MWCNTs). The study explores the potential applications of these nanocomposites in various engineering fields where friction and wear resistance are crucial factors. The following factors present the novelty of this manuscript.

Nanocomposite Fabrication: This manuscript describes a novel approach for fabricating the PMMA/MWCNT nanocomposites via a 3D mixing approach. This approach involves optimizing the processing parameters, such as the dispersion method and mixing technique, to achieve a homogeneous distribution of MWCNTs within the PMMA matrix. Tribological testing includes the development of custom-designed test specimens to measure frictional forces, wear rates, and surface morphology changes during testing.

Wear Characterization: The manuscript focuses on a wear characterization approach to evaluate the wear mechanisms and wear resistance of the PMMA/MWCNT nanocomposites, using scanning electron microscopy (SEM) to analyze the worn surfaces and quantify wear parameters like wear volume, wear rate, or the coefficient of friction.

Performance Evaluation: The reported work presents a comprehensive performance evaluation of the PMMA/MWCNT nanocomposites, comparing their friction and wear properties with those of existing materials or conventional PMMA composites. The study highlights the superior tribological performance of the nanocomposites and discusses the underlying mechanisms responsible for the observed improvements.

## 2. Materials and Methods

### 2.1. Materials

Polymethyl methacrylate (PMMA)

The polymerization of methyl methacrylate leads to the development of artificial resin PMMA, popularly known as acrylic. 

Multi-wall Carbon Nanotubes (MWCNT)

MWCNTs functionalized by two different groups (hydroxyl and carboxyl) and synthesized via catalytic CVD processes, with a high purity of 99%, diameters of 12 to 15 nm, and ultra-high aspect ratios, obtained from Autus Nanolab PVT. Ltd., were used in this study.

### 2.2. Specimen Preparation

**Figure 1:**
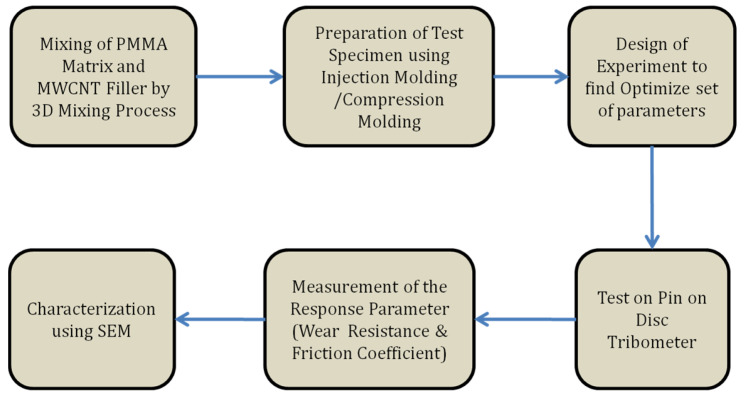
Schematic flow chart for experimental process.

With the use of a twin-screw extruder and a 3D mixture, PMMA and MWCNT were compounded. Before compounding, the PMMA was dried for six hours at 60 °C in the oven to eliminate moisture. The extruder’s barrel temperatures were regulated zone by zone for proper material flow. Throughout the procedure, the extruder’s die temperature was regulated to 280 °C. The extruded material was cooled using a water bath, and the string of nanocomposites was transformed into pellets at the other end using a pelletizer. The composite pellets were again dried at 60 °C for 6 h following extrusion to eliminate moisture. Injection molding was used to create dried pellets in the shape of specimen pins for tribological testing. As shown in Figure 1, the specimens were prepared filler contents of 0.1, 0.5, and 1 wt.% of MWCNT to determine the effect on the tribological properties of the PMMA matrix material. Figure 1 shows the methodology.

### 2.3. Test Procedure

#### Pin-on-Disk Tribological Test

**Figure 2:**
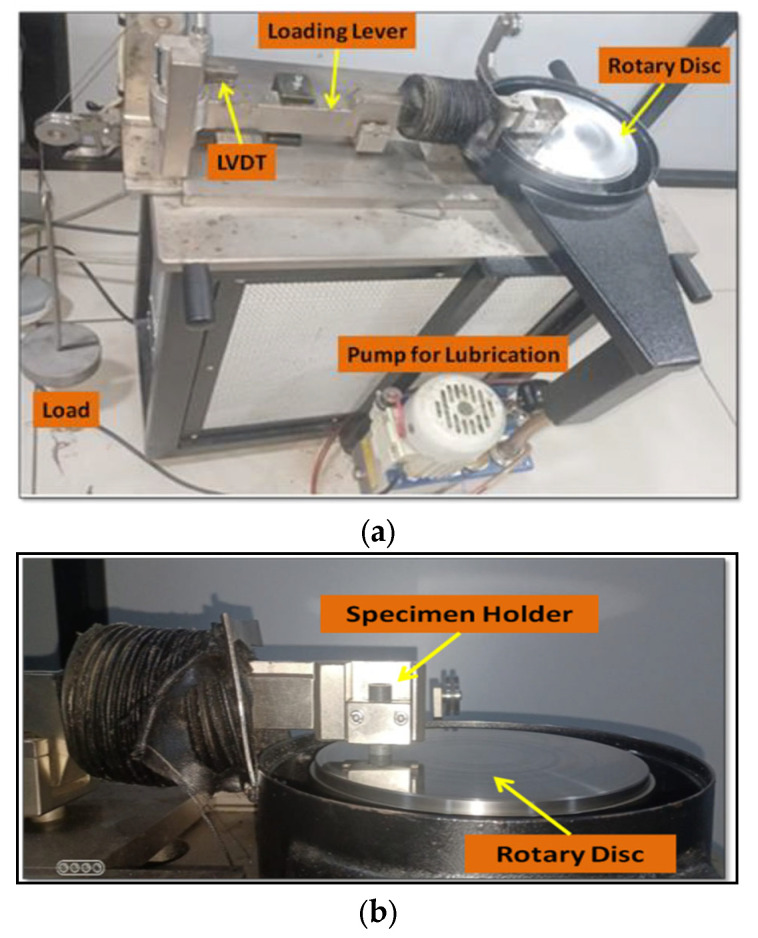
Tribology testing apparatus: (**a**) pin-on-disk setup (**b**) specimen on hardened EN31 steel disc.

On a DUCOM TR-20LE-PHM-200 pin-on-disk tribometer operating at room temperature, friction and wear tests involving sliding the specimen against a pin on a hardened EN31 steel disk were carried out. Figure 2a,b show the friction couple’s schematic diagram. The disk or specimen is pinched around the disc’s center by the testing device. In either case, the sliding path on the disc’s surface is circular. Usually, a weighted arm or lever with a predetermined load is used to press the pinned specimen against the disc. To obtain wear data, the test must be run with a predetermined sliding distance, weight (20, 30, and 60 kg), and speed (958 RPM). A sample with a diameter of 10 mm and a length of 32 mm was created for this experiment, and the procedures described above were used to achieve the desired outcome. All tests were conducted at a temperature of 27 °C and a track diameter of 100 mm. The speed and load were chosen considering the ASTM G99 requirements. An electronic sensor fitted to the machine continually measured and logged the friction and wear coefficients.

### 2.4. Scanning Electron Microscopy

Scanning electron microscopy (SEM) is a powerful technique for investigating wear mechanisms and analyzing wear surfaces at micro- and nanoscales. Studying particle dispersion and agglomeration at the interphase were the main objectives of utilizing SEM. Images were taken of the processed nanocomposite material to validate the morphological changes in the microstructures of the composites produced with the inclusion of MWCNTs. Small sections of the samples were subjected to scanning electron microscopy (SEM) utilizing a low-voltage type of SEM, a JEOL JSM-7010LA (JEOL Ltd., Tokyo, Japan). To examine the microstructures of the nanocomposites, cracked surfaces are most frequently used. The lack of conductivity in polymer nanocomposites is a problem. The nonconducting surfaces of the composites were coated with gold via an agar auto-sputter coater to induce conductivity before they were examined from microstructural point of view.

### 2.5. Design of Experiments

In the present research, RSM was selected for wear loss to determine the relationship between the applied loads, reinforcement wt.%, and the track distance of the PMMA/MWCNT composites. The three input process variables and their three levels required 27 tests, as shown in Table 2. The design was created and analyzed using design expert software. The parameters of all 27 trials, as shown in Table 3, combined and performed experimentation results.

## 3. Results and Discussions

The functionalization of multi-walled carbon nanotubes (MWCNTs) can have a significant impact on the wear properties of the nanotubes and the materials into which they are incorporated. Hydroxyl-functionalized MWCNTs exhibit improved lubrication, reduced friction, enhanced load-bearing capacities, and resistance to wear and degradation, making them advantageous for various tribological applications, including lubricants, coatings, and solid lubricant additives.

### 3.1. Wear Resistance

In this research, the authors used the ANOVA–quadratic model, which is an extension of the traditional ANOVA model that incorporates quadratic terms to account for non-linear relationships. It detects and analyzes non-linear relationships between variables; it can also provide a better fit to the data compared to a linear model, leading to more accurate predictions and more reliable statistical inferences. This method also enables hypothesis testing on the significance of the quadratic terms, providing statistical evidence for non-linear relationships. This allows researchers to determine whether the inclusion of quadratic terms significantly improves the model fit and justifies the use of a more complex model. The ANOVA–quadratic model offers several advantages over the traditional ANOVA model by allowing for the analysis of non-linear relationships and providing a better fit to the data.

Understanding the *p*-value coefficient and comparing it with our significance-level value (usually 0.05) is essential for determining whether a statistically significant relationship between the response and predictors is seen. The *p*-values obtained for the current work are shown in Table 4. There is a substantial correlation between the predictors and response since all *p*-values are less than 0.005.

Given the model’s *F*-value of 136.28, the model is probably significant. For just 0.01% of the time might an *F*-value this large be caused by noise. When the *p*-value is less than 0.0500, the model terms are deemed significant. The model terms A, B, C, AC, A2, B, and C2 are crucial. If the value is higher than 0.1000, the model terms are not significant.

Table 5 shows a discrepancy of less than 0.2 between the Predicted R2 of 0.9623 and the Adjusted R2 of 0.9791. Adeq Precision also measures the signal-to-noise ratio. The ideal ratio is at least 4. A ratio of 42.147 indicates a sufficient signal. This model is used to navigate the design area.

**Figure 3:**
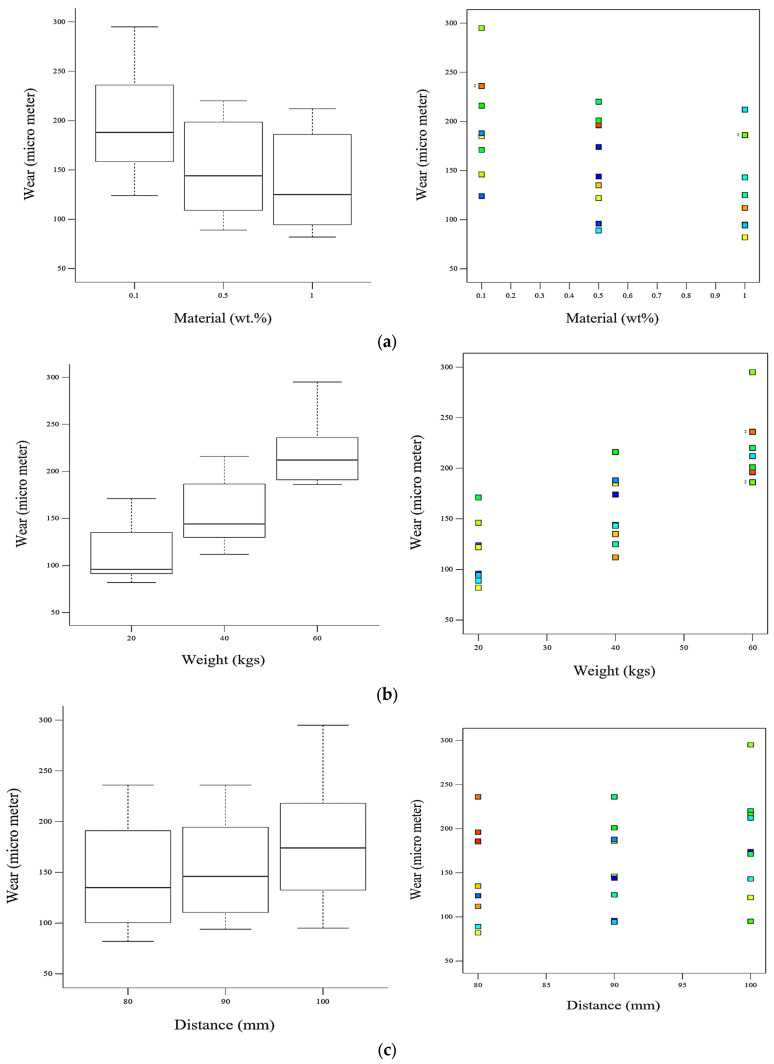
Effect of an individual factor on the response, shown by scatter and box plots for: (**a**) wt.% of nanofiller material; (**b**) weight/load; (**c**) track distance.

The scatter and box plots of the wear and variable inputs are shown in Figure 3. Weight significantly impacts wear, as the wear vs. weight scatter and box plots show. An increase in weight causes a more significant increase in wear than other input parameters. The increase in the material’s weight caused a decrease in the wear of the polymer nanocomposite material. From the scatter and box plots of wear vs. distance, an increase in the space slightly increased the wear.

**Figure 4:**
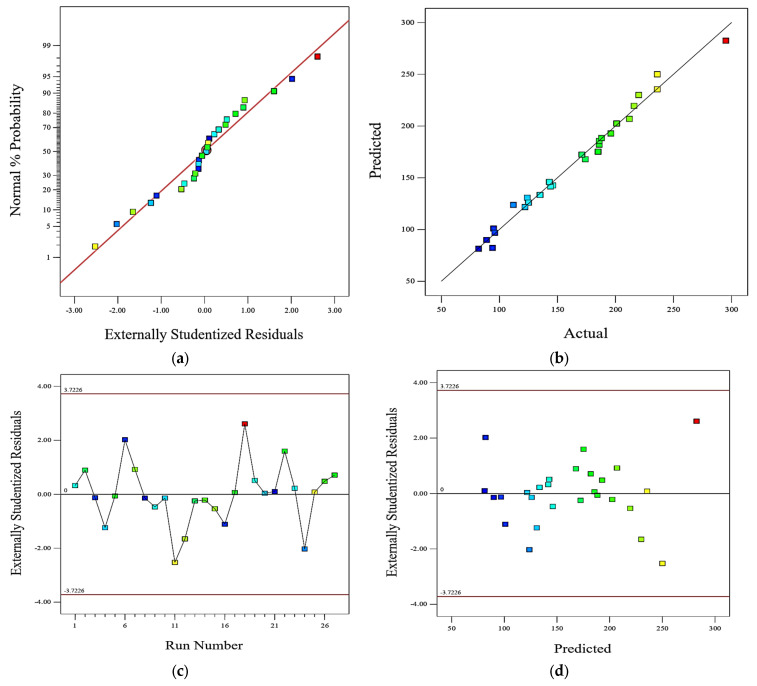
Graph of: (**a**) normal probability plot on wear loss; (**b**) actual vs. predicted values; (**c**) residuals vs. runs; (**d**) residuals vs. predicted.

Figure 4 provides visual representations of the relationships between residuals and various factors such as normal probability, actual values, run order, and predicted values. The observations made from these graphs suggest that the residuals are normally distributed, the predictions are close to the actual values, and all the experiments fall within the acceptable range of residuals.

Figure 4a shows the relationship between the residuals (the differences between the actual values and the predicted values) and the normal probability. The normal probability represents how likely it is for the residuals to follow a normal distribution. It can be observed that the residuals tend to cluster around the regression line, indicating that they are normally distributed.

Figure 4b displays the relationship between the predicted values and the actual values. The experiments or data points fall close to the diagonal line, which suggests that the predicted values are close to the actual values. In this case, the experiments lie around the regression line, indicating that the predictions are reasonably accurate.

Figure 4c,d represent the relationships between the residuals and the run order (the order in which the experiments were conducted) and the residuals and the predicted values, respectively. In both graphs, the range of the residuals is considered by comparing them to the minimum and maximum limits. By examining these graphs, it can be observed that none of the experiments exceed these limits, indicating that all the experiments’ residuals are within an acceptable range.

**Figure 5:**
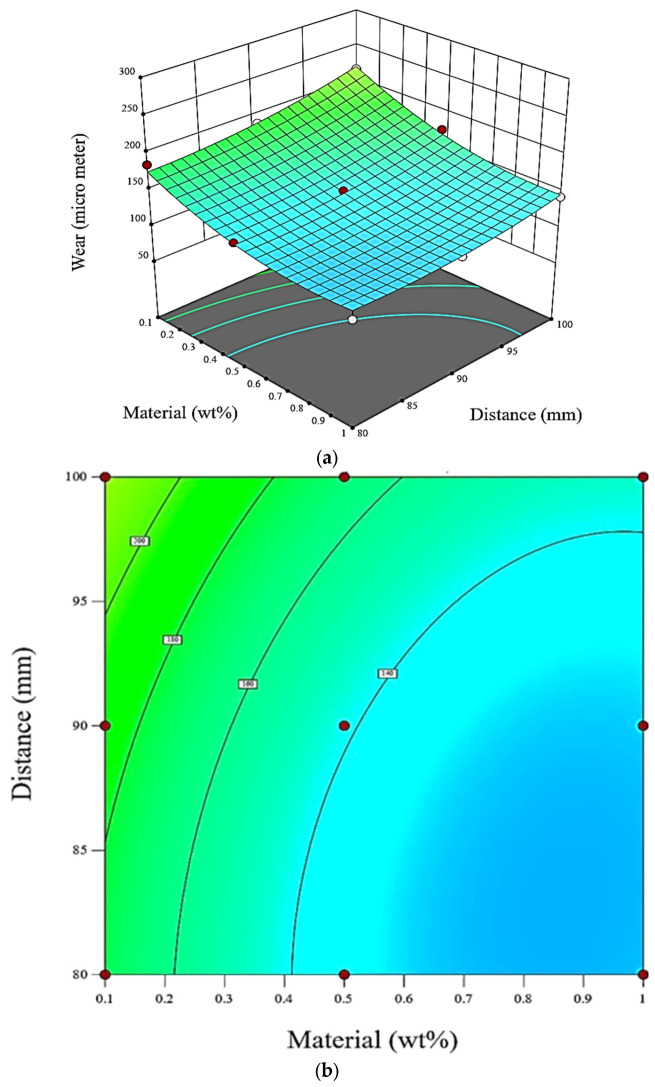
Graph for specific wear rate as a function of filler content and track distance: (**a**) 3D surface; (**b**) 2D contour plot.

In Figure 5, the relationships between filler content, track distance, and wear rate are visually represented using 3D surface and contour plots. These plots provide a comprehensive view of how these factors interact and influence the wear rate. The data used to create these graphs were obtained from experiments conducted using Design Expert software, and a regression equation was derived from these data to accurately depict the relationship between the design factors and the wear rate.

The 3D surface plot (Figure 5a) shows the wear rate as a function of the filler content and track distance. The two axes represent the values of these factors, while the vertical axis represents the wear rate. The surface plot provides a three-dimensional visualization of how changes in filler content and track distance impact the wear rate.

The contour plot depicted in Figure 5b, on the other hand, represents the wear rate through contour lines on a two-dimensional plane. The contours connect points with equal wear rate values, allowing us to identify regions of similar wear rate levels. By examining the contour lines, one can observe how changes in the filler content and track distance influence the wear rate and identify areas of higher or lower wear rates.

In the graphs marked with the middle level of one parameter held constant while the other two parameters were changed, the aim was to isolate the effect of each factor and observe its impact on the wear rate. In fixing one parameter at a specific value and varying the other two, the relationship between the fixed parameter and the wear rate becomes more evident. This analysis helped us understand the individual contributions of filler content and track distance to the wear rate.

Based on the observations from these plots, it can be concluded that a decrease in the relative weight of the material (represented by a decrease in the filler content) and an increase in the distance travelled along the track both lead to an increase in the wear rate. This implies that reducing the filler content and increasing the track distance will result in higher wear rates. These findings provide valuable insights into the relationship between the design factors and the wear rate, allowing for informed decision making in optimizing the parameters to minimize wear.

### 3.2. Friction Coefficient Results

The model’s significance, the significance of specific model coefficients, and the lack of a fit test must all be analyzed via the ANOVA, using the nominal model terms in the ANOVA by choosing the backward elimination approach. The ANOVA, Table 6, showed that the composites’ reinforcing weight percentage, load, and track length affected the friction coefficient. The final quadratic model for the friction coefficient in terms of the coded components is their graphical relation with different factors in the section below.

The model is suggested to be significant by the model’s *F*-value of 296.40. An *F*-value this large might happen due to noise less than 0.01% of the time. Model terms are considered significant when the *p*-value is less than 0.0500. Important model words are Material, Weight, Distance, Material∗Weight, and Second Order of Material. Model terms are not significant if the value is higher than 0.1000. Model reduction may enhance your model if it has many unnecessary words (except those needed to maintain a hierarchy).

According to Table 7, the discrepancy between the Predicted R2 of 0.9937 and the Adjusted R2 of 0.9903 is less than 0.2. Adeq Precision measures the ratio of signal to noise. A ratio of at least four is preferred. A sufficient signal is indicated by a percentage of 54.2742.

**Figure 6:**
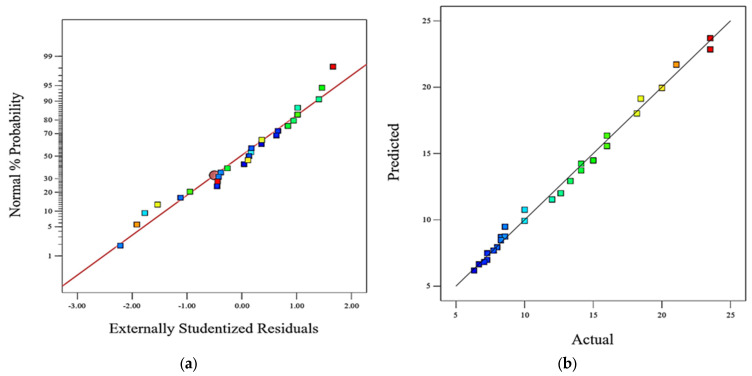

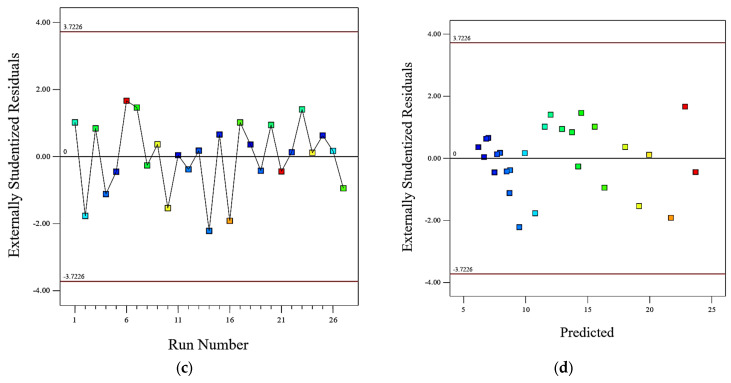
Graph of: (**a**) normal probability plot on friction force; (**b**) actual vs. predicted values; (**c**) residuals vs. runs; (**d**) residuals vs. predicted values.

Figure 6 provides evidence that the statistical model used is valid and reliable. The experiments align with the regression line, indicating a good fit between the predicted and actual values. Additionally, the residuals remain within the acceptable range, demonstrating that the model accurately captures the variation in the data. These findings support the quality and accuracy of the statistical analysis performed. Figure 6a depicts the residuals (the discrepancies between observed and anticipated values) that are displayed in a normal probability plot. The residuals cluster close to the regression line, as can be seen. This is promising in statistical modelling since it implies that the residuals have a normal distribution. The graph’s points in Figure 6b represent the experiments or data points clustered along the regression line. This suggests that the statistical model provides an excellent fit, as the anticipated values are in close agreement with the observed values. Figure 6c,d suggest that the residuals fall within an acceptable range and there are no extreme outliers. It indicates that the model effectively captures the variability in the data without any unusual or problematic observations.

**Figure 7:**
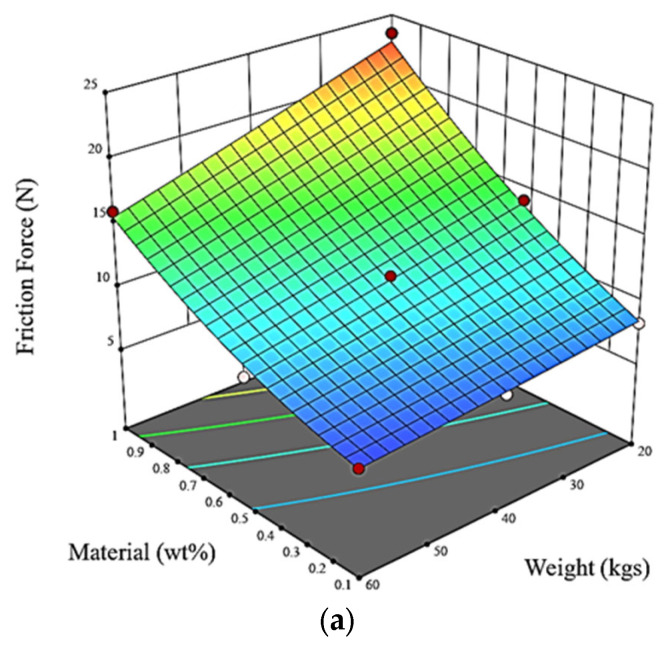

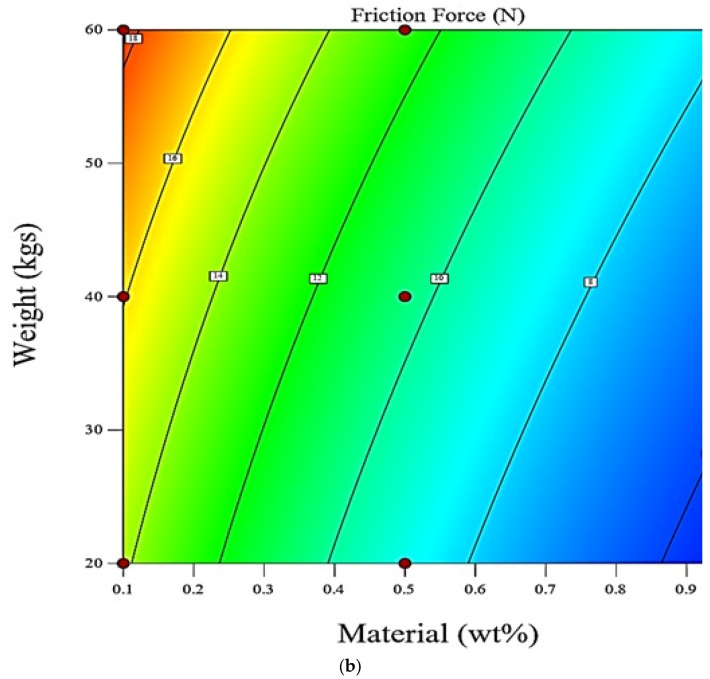
Graphs for friction force as a function of filler content and weight: (**a**) 3D surface; (**b**) 2D contour plot.

Figure 7 employs 3D surface and 2D contour plots to depict the impacts of material and weight on friction force. The regression equation derived from the experimental data allows for the visualization of the relationship between friction force and the design elements. The findings indicate that increases in the mass and density of the materials correspond to higher frictional resistance. These insights offer valuable information for understanding and optimizing frictional behavior in the given system. Using Design Expert software, the experimental data were utilized to develop a regression equation that enabled the generation of graphs illustrating the relationship between friction force and the design factors.

It can be observed from Figure 7a,b that increases in the mass and density of the materials lead to a corresponding increase in frictional resistance. This implies that as the material becomes heavier and denser, it generates higher levels of frictional force. This observation suggests that material and weight are significant factors influencing the friction force.

Moreover, the functionalization of multi-walled carbon nanotubes (MWCNTs) can have a significant impact on the wear properties of the nanotubes and the materials into which they are incorporated. Hydroxyl-functionalized MWCNTs show enhanced tribological results. They also exhibit improved lubrication, reduced friction, enhanced load-bearing capacities, and resistance to wear and degradation, making them advantageous for various tribological applications, including their use as lubricants, coatings, and solid lubricant additives.

### 3.3. Scanning Electron Microscopy

**Figure 8:**
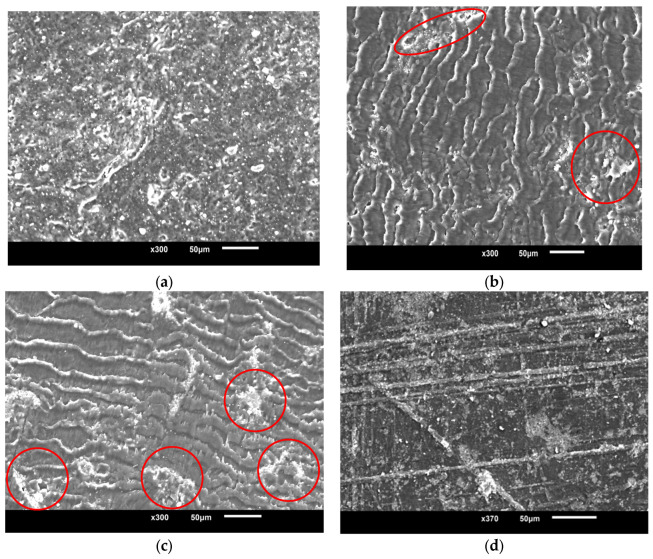
SEM micrographic of MWCNT-reinforced PMMA composite surface: (**a**) 0.1 wt.% of MWCNT in PMMA; (**b**) 0.5 wt.% of MWCNT in PMMA; (**c**) 1.0 wt.% of MWCNT in PMMA; (**d**) randomly oriented MWCNTs in PMMA matrix.

Regarding the functional properties of MWCNT-reinforced nanocomposites, the dispersion and alignment of the nanotubes inside a polymer matrix are two of the most important factors. In this context, the authors began by employing scanning electron microscopy (SEM) to characterize the dispersion and alignment of the nanotubes. Figure 8 demonstrates the scanning electron micrograph pictures of the cross-section of the nanocomposite. These images validate the dispersion and alignment of nanotubes within the PMMA matrix. These images suggest that nanotubes are evenly distributed throughout the polymer phase and that the orientation of the nanotubes is parallel to the direction of the shear flow, for which the flow direction is indicated by an arrow in the micrograph. Because of the significant shear stress that is present in the direction that the melt flows during the injection molding process, the CNTs contained inside the nanocomposites become partially aligned with the flow direction.

In addition, considering that the injected specimens were cut at an angle that was perpendicular to the flow direction and that the heads of the CNTs appeared to be facing the flow direction within the PMMA matrix in Figure 8, one can deduce that the orientation of the nanotubes is in the direction of the shear flow. Figure 8d shows the random orientation of MWCNTs in the PMMA matrix, and no agglomeration or a very small agglomeration was found in the micrograph. The agglomeration has been indicated in Figure 8 with red circles

### 3.4. Future Scope

The field of polymer nanocomposite tribology is expected to continue advancing and evolving. Tribology has been playing a crucial role in various industries, including the automotive, aerospace, manufacturing, and energy industries. A focus on designing novel nanocomposite materials with tailored properties to improve tribological performance is suggested. Moreover, surface modifications and coatings are essential for reducing friction and wear in tribological systems. Nanocomposite coatings, consisting of nanoparticles embedded in a suitable matrix, can offer improved wear resistance, reduced friction, and enhanced lubrication properties. As sustainability and environmental concerns continue to grow, future research in nanocomposite tribology will focus on developing eco-friendly and energy-efficient solutions.

Moreover, pin-on-disc (POD) testing is a widely used tribological test method, but it does have some limitations. The POD test assumes a simplified contact geometry between the pin and the disc, which may not accurately represent the actual contact conditions in real-world applications. Real-world tribological systems often involve variable loads, speeds, and directions which can significantly affect wear behavior, which is missing in the POD test. There are few alternative methods for tribological tests: reciprocating or oscillating tribometers, block-on-ring tribometers, in situ tribological testing, and real-world application testing.

## 4. Conclusions

In the current study, we investigated how carbon nanotubes affect the tribological characteristics of nanocomposite materials. The impact of MWCNTs at various mixture ratios was studied using a pin-on-disk test apparatus. The study initially focused on the tribological behavior and the development of friction layers during tribological experiments on these novel materials.

The key findings may be summarized as follows:

- The track distance and the mixing ratio significantly influence the wear characteristics, the mixing ratio, the load (weight), and the high-impact friction qualities.
- The more significant the matrix to MWCNT mixing ratio utilized in material preparation is, the greater the friction coefficient and wear rate will be.
- It was found that the functionalized MWCNTs promote load transfer and interfacial adhesion between the nanotubes and PMMA matrix, improving mechanical integrity and wear resistance.
- The results determined the appropriate MWCNT loading concentration for friction and wear on a PMMA matrix. Beyond a specific level, nanotube loading may cause agglomeration or inefficient dispersion, reducing performance.
- The inclusion of functionalized MWCNTs affects the primary wear mechanisms, such as abrasion, adhesion, and tribochemical reactions, reducing wear volume and enhancing wear resistance.

## Figures and Tables

**Table 1:** Tribology Characteristics of polymer nanocomposites.

Sr. No	Material	Test Method	Properties/Results	Ref.
1	PEEK/SCF/zirconia	Pin-on-disc test	Excellent wear resistance under aqueous conditions.	[19]
2	Carbon nanofibres/ PEEK	Unidirectional sliding tests	Reduces the wear rate of PEEK very significantly	[20]
3	High-density poly(ethylene) (HDPE)/MWCNT	Block-on-ring test	Wear resistance and frictional properties of HDPE were found to improve in the presence of the nanofillers.	[21]
4	PMMA/calcium carbonate (CaCO_3_) nanoparticles	Pin-on-disk tribometer	Significantly improved the abrasion resistance of PMMA.	[22]
5	PMMA/silica nanoparticles	Pin-on-disk test	Improved the abrasion resistance of PMMA.	[23]
6	PMMA/PS/MWNTs	Pin-on-disk test under dry conditions	Better wear resistance but also a smaller friction coefficient.	[24]
7	Poly(ethylene) terephthalate (PET) filled with alumina nanoparticles	Pin-on-disk test	Increased the wear resistance.	[25]
8	PPS + SWCNT	End-face contact tribometer	Improvements in wear resistance and friction coefficient.	[26]
9	Nylon 6/ 5 wt.% nanoclay nanocomposite	Pin-on-disk test	Wear rate increases drastically and is much higher than the wear rate of the neat polymer.	[27]
10	PTFE/ graphene	Unidirectional sliding tests	Lower wear rates could be achieved with higher graphene platelet concentration.	[27]

**Table 2:** Parameters and their levels.

Sr. No	Variables	A	B	C
1	Material (wt.%)	0.1	0.5	1.0
2	Load (Weight) (Kgs)	20	30	60
3	Track Diameter (mm)	60	90	100

**Table 3:** L27 array for a combination of parameters and results.

	Factor 1	Factor 2	Factor 3	Response 1	Response 2
Run	A: Material	B: Load (Weight)	C: Track Diameter (T.D.)	Wear	Friction Force
	wt.%	Kg	mm	Micrometer	*n*
1	0.5	30	90	144	12.0
2	0.5	30	100	174	10.0
3	0.5	20	90	96	14.1
4	0.1	20	60	124	8.3
5	0.1	30	90	188	7.3
6	1	20	90	94	23.5
7	1	60	100	212	15.0
8	0.5	20	60	89	14.1
9	1	30	100	143	18.2
10	1	30	90	125	18.5
11	0.1	60	90	236	6.7
12	0.5	60	100	220	8.6
13	0.1	20	100	171	8.0
14	0.5	60	90	201	8.6
15	0.1	30	100	216	7.3
16	1	20	100	95	21.1
17	1	60	90	186	16.0
18	0.1	60	100	295	6.3
19	0.1	20	90	146	8.3
20	0.5	20	100	122	13.3
21	1	20	60	82	23.5
22	0.1	30	60	185	7.7
23	0.5	30	60	135	12.6
24	1	30	60	112	20.0
25	0.1	60	60	236	7.1
26	0.5	60	60	196	10.0
27	1	60	60	186	16.0

**Table 4:** ANOVA–quadratic model for wear resistance.

Source	Sum of Squares	Degree of Freedom (df)	Mean Square	Fit Summary (*F*-Value)	*p*-Value-Coefficient	
Model	75,370.95	9	8374.55	136.28	<0.0001	significant
Material	17,546.89	1	17,546.89	285.54	<0.0001	significant
Weight	49,859.51	1	49,859.51	811.36	<0.0001	significant
Distance	4967.31	1	4967.31	80.83	<0.0001	significant
Material∗Weight	12.55	1	12.55	0.2042	0.6571	
Material∗Distance	366.40	1	366.40	5.96	0.0258	significant
Weight∗Distance	21.33	1	21.33	0.3472	0.5635	
Material^2^	2137.54	1	2137.54	34.78	<0.0001	significant
Weight^2^	378.69	1	378.69	6.16	0.0238	significant
Distance^2^	480.02	1	480.02	7.81	0.0124	significant
Residual	1044.68	17	61.45			
Cor Total	76,415.63	26				

**Table 5:** Fit statistics.

Std. Dev.	7.84	R²	0.9863
Mean	163.30	Adjusted R²	0.9791
C.V. %	4.80	Predicted R²	0.9623
		Adeq Precision	42.1468

**Table 6:** ANOVA–quadratic model for friction coefficient.

Source	Sum of Squares	df	Mean Square	*F*-Value	*p*-Value	
Model	750.41	9	83.38	296.40	<0.0001	significant
Material	611.04	1	611.04	2172.19	<0.0001	significant
Weight	93.01	1	93.01	330.66	<0.0001	significant
Distance	7.76	1	7.76	27.59	<0.0001	significant
Material∗Weight	22.54	1	22.54	80.14	<0.0001	significant
Material∗Distance	1.14	1	1.14	4.04	0.0607	
Weight∗Distance	0.0111	1	0.0111	0.0395	0.8447	
Material^2^	7.60	1	7.60	27.01	<0.0001	significant
Weight^2^	0.0309	1	0.0309	0.1098	0.7445	
Distance^2^	0.1358	1	0.1358	0.4826	0.4966	
Residual	4.78	17	0.2813			
Cor Total	755.19	26				

**Table 7:** Fit statistics.

Std. Dev.	0.5304	R²	0.9937
Mean	12.67	Adjusted R²	0.9903
C.V. %	4.19	Predicted R²	0.9842
		Adeq Precision	54.2742

## Data Availability

Not applicable.
